# Production of Polyhydroxyalkanoates in Unsterilized Hyper-Saline Medium by Halophiles Using Waste Silkworm Excrement as Carbon Source

**DOI:** 10.3390/molecules26237122

**Published:** 2021-11-25

**Authors:** Shuangfeng Cai, Yaran Wu, Yanan Li, Shuying Yang, Zhi Liu, Yuwen Ma, Jianqiang Lv, Yujia Shao, Hongzhe Jia, Yan Zhao, Lei Cai

**Affiliations:** 1 Engineering Research Centre of Molecular Medicine of Ministry of Education, Key Laboratory of Fujian Molecular Medicine, Key Laboratory of Precision Medicine and Molecular Diagnosis of Fujian Universities, Key Laboratory of Xiamen Marine and Gene Drugs, School of Biomedical Sciences and School of Medicine, Huaqiao University, Xiamen 361021, China; caisf@hqu.edu.cn (S.C.); yananli12@163.com (Y.L.); 15856235280@139.com (S.Y.); 2 School of Food Science and Biotechnology, Zhejiang Gongshang University, Hangzhou 310035, China; yaransun@163.com (Y.W.); liuzhi021019@outlook.com (Z.L.); myw011024@163.com (Y.M.); lvyes3838@163.com (J.L.); xxx953670196@163.com (Y.S.); lukebella0424@163.com (H.J.); Yanzhao9918@163.com (Y.Z.)

**Keywords:** polyhydroxyalkanoate, halophiles, endogenous microorganisms, silkworm excrement, open fermentation

## Abstract

The chlorophyll ethanol-extracted silkworm excrement was hardly biologically reused or fermented by most microorganisms. However, partial extremely environmental halophiles were reported to be able to utilize a variety of inexpensive carbon sources to accumulate polyhydroxyalkanoates. In this study, by using the nile red staining and gas chromatography assays, two endogenous haloarchaea strains: *Haloarcula hispanica* A85 and *Natrinema altunense* A112 of silkworm excrement were shown to accumulate poly(3-hydroxybutyrate) up to 0.23 g/L and 0.08 g/L, respectively, when using the silkworm excrement as the sole carbon source. The PHA production of two haloarchaea showed no significant decreases in the silkworm excrement medium without being sterilized compared to that of the sterilized medium. Meanwhile, the CFU experiments revealed that there were more than 60% target PHAs producing haloarchaea cells at the time of the highest PHAs production, and the addition of 0.5% glucose into the open fermentation medium can largely increase both the ratio of target haloarchaea cells (to nearly 100%) and the production of PHAs. In conclusion, our study demonstrated the feasibility of using endogenous haloarchaea to utilize waste silkworm excrement, effectively. The introduce of halophiles could provide a potential way for open fermentation to further lower the cost of the production of PHAs.

## 1. Introduction

The massive use of traditional, nonbiodegradable plastics results in severe environmental pollution and the large consumption of nonrenewable fossil resources [1]; biodegradable plastics have become a hotspot of concern in the world today. Polyhydroxyalkanoates (PHAs) are biodegradable polyesters produced to store excess carbon sources in cells when the microbial growth lacks nitrogen, and phosphorus sources and carbon sources are sufficient [2]. In contrast to traditional petroleum-based plastics, PHAs have similar material properties but are completely degradable in a natural environment and, also, have many advantages, such as biocompatibility, a gas barrier, etc. [3]. They can be used as medical materials without causing adverse reactions in humans, such as bone nails, vascular stents, and so on [4]. At present, the production of PHAs still has many limitations; for instance, their cost is higher than that of traditional plastics. The carbon source is the key to the synthesis of PHAs, but the cost of carbon sources in the fermentation accounts for about half of the total cost [5], which seriously hinders the large-scale production and application of PHAs in the industry.

*Bombyx mori* (silkworm) take mulberry leaves as their main food. The feces of *Bombyx mori*, referred in this study as the silkworm excrement (SE), is a typical agricultural waste in the sericulture industry in China [6]. Since the gastrointestinal tract of *Bombyx mori* is very inefficient in digesting mulberry leaves, silkworm excrement is nutritious, containing different crude proteins, polysaccharides, crude fibers, chlorophyll, pectin, and other active ingredients [7]. However, owing to the growth characteristics of the mulberry trees, it contains high concentrations of cadmium, arsenic, and other heavy metals [8], which is unfavorable to be used as an agricultural fertilizer, so most of it is discarded and has low bioavailability. This is not only a waste of resources but also causes environmental pollution. With the development of science and technology, the functional active ingredients in silkworm excrement have been studied. In particular, the production of chlorophyll from silkworm excrement has been well-developed industrially. Han et al. optimized the simultaneous aqueous two-phase extraction and saponification reaction to extract chlorophyll sodium salt from silkworm excrement, which developed a simple method for the extraction of chlorophyll sodium salt [9]. However, the reuse of chlorophyll extracted silkworm excrement has rarely been reported.

Halophilic microorganisms are a group of microorganisms that grow in high-salt environments. They mainly grow in environments with high salt concentrations like salt lakes and salt farms or salt-cured products like pickles [10]. PHA-producing strains have been identified in a variety of halophilic microorganisms, including the genera *Halomonas* [11], *Natrinema*, *Haloferax*, and *Halococcus* [12]. Halophilic microorganisms can not only accumulate PHAs using expensive carbon sources like glucose but also synthesize PHAs with high yields using inexpensive carbon sources such as corn stover and whey [11]. Two halophilic archaea, *Natrinema altunense* and *Haloterrigena jeotgali*, screened from a salt lake could use industrial sugar wastewater as a carbon source for the fermentation of PHA production [13]. Kucera et al. found that the strain *Halomonas halophila* can synthesize PHAs well using industrial inexpensive carbon sources such as cheese whey, spent coffee grounds, sawdust, and corn stover, accumulating up to 82% of the cell dry weight [11]. Among them, poly(3-hydroxybutyrate) (PHB) and poly(3-hydroxy-butyrate-*co*-3-hydroxyvalerate) (PHBV) have been the most studied. Compared with PHB, PHBV has better strength, hardness, greater elasticity, and lower melting point [14,15]. Up to now, all wild-type PHBV-producing bacteria required additional expensive carbon sources with odd-numbered short-chain fatty acid such as propionic acid or valeric acid, but most of the haloarchaea can accumulate PHBV by directly adding inexpensive unrelated carbon sources [16]. For example, the strain *Haloferax mediterranei* can use olive mill wastewater as the only carbon source to accumulate PHBV (containing 6% 3-hydroxyvalerate (3-HV)) [17]. Therefore, it is important to study the synthesis of PHA by halophiles using inexpensive carbon sources. Meanwhile, the introduction of halophiles could provide a potential way for open fermentation, and in addition, the fermentation of halophilic bacteria is beneficial to the cell lysis in water exposed to extract PHAs, giving it an advantage in the competition with other microorganisms [18].

Studies of open fermentation are needed to lower the cost of equipment sterilization in the industrial production of PHAs [19]. Currently, there are many reports on open fermentation processes. Munir et al. studied the production of PHA from waste-activated sludge by open fermentation of mixed bacteria using different fatty acids such as acetic acid, with yields up to 0.32 gPHA gVSS^−1^ [20]. The high NaCl concentration of the medium makes the fermentation process possible without sterilization [18]. Tan et al. successfully developed the production of PHB by open, continuous fermentation using the strain *Halomonas* TD01, which reduced the cost of aseptic fermentation [21]. It has been shown that the halophilic bacterium *Halomonas campaniensis* LS21 can accumulate PHB by open fermentation using mixed substrates such as food waste for 65 days, demonstrating the great potential of halophilic open fermentation [22].

In order to lower the cost for PHA production, a lot of research has been conducted on whey, waste oils, molasses, etc. [23]. However, the utilization of silkworm excrement has not been reported yet. Our previous experiments found that the well-known PHA-accumulating bacteria reported, such as the strains *Ralstonia eutropha* H16 and *Halomonas venusta*, could not grow when using silkworm excrement as the sole carbon source [24]. Therefore, this study attempted to independently isolate and screen the endogenous halotolerant PHAs accumulating microorganisms from the silkworm excrement and investigate their open fermentation production characteristics when using chlorophyll ethanol-extracted silkworm excrement as the sole or partial carbon source, which can not only improve the PHA synthesis ability and reduce the cost of PHA synthesis but also realize waste recycling.

## 2. Results

### 2.1. Isolation and Identification of the Endogenous Halotolerant Microorganisms in Silkworm Excrement

Our previous study showed that a culture medium with less than 10% sodium chloride concentration cannot inhibit the growth of most endogenous microorganisms in silkworm excrement [24], while a medium with 15% sodium chloride concentration can. Two promised PHA-producing strains: *Ralstonia eutropha* H16 and *Halomonas venusta* were shown to not be able to produce PHA from silkworm excrement [24]. Therefore, in this study, for screening the candidate strains for the open fermentation with non-sterilized silkworm excrement as the carbon source, the isolation and identification of endogenous microorganisms from silkworm excrement were carried out by using AS-165 with a 15% sodium chloride concentration as the medium, and 16S rDNA identification was performed on all the different colonies isolated from the silkworm excrement. The results showed that a total of 14 different microorganisms were isolated, of which 10 were bacteria and four were archaea (Table 1). All the strains obtained were 100% identical in the 16S rDNA sequence to the identified strains in the database, except for the strains *Halorubrum aidingense* A28 (99.88%), *Haloarcula hispanica* A85 (99.72%), *Halomonas janggokensis* B6 (98.89%), and *Brachybacterium paraconglomeratum* BZ6 (99.72%).

### 2.2. Screening of Endogenous PHAs Accumulating Strains in Silkworm Excrement

**Figure 1:**
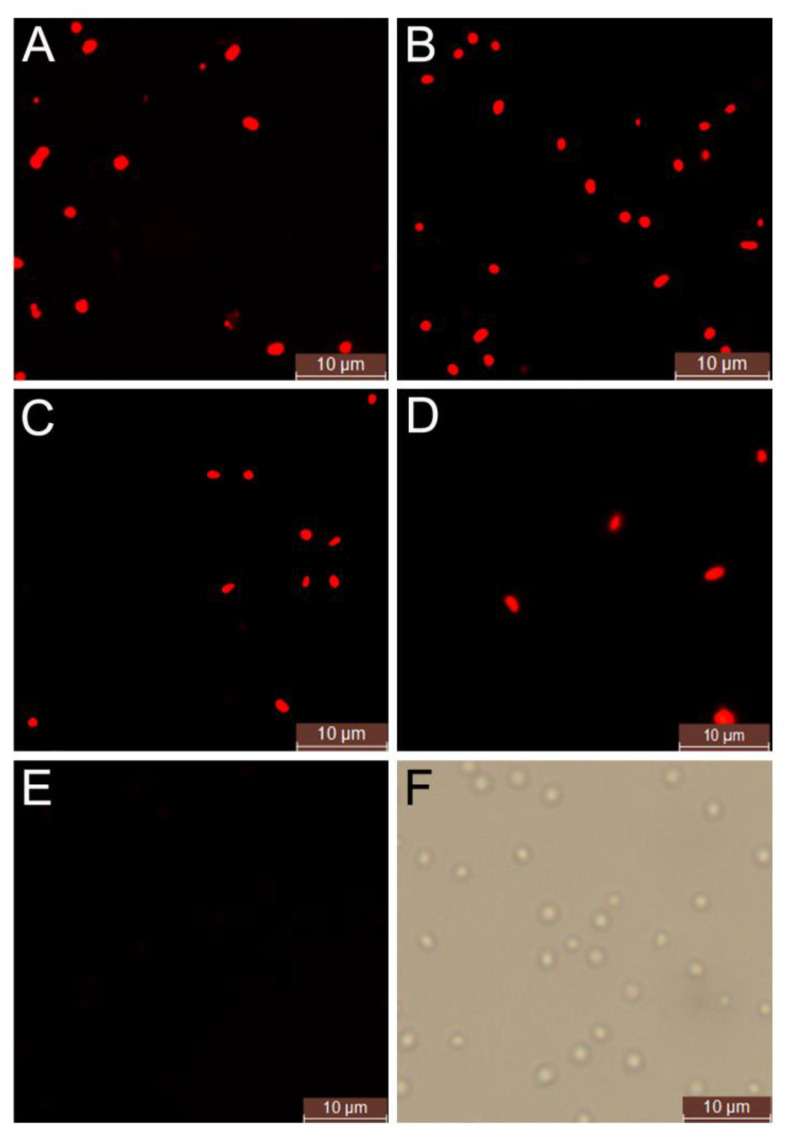
The fluorescence microscope examination of endogenous PHA-accumulating strains *Haloarcula hispanica* A85 (**A**), *Natrinema altunense* A112 (**B**), *Halomonas salina* BSF4 (**C**), and *Halomonas janggokensis* B6 (**D**) stained with Nile red. The Nile red-stained PHA-defeated strain *Haloferax mediterranei* ΔEC was used as a negative control to preliminarily determine the accumulation of PHAs observed under fluorescence (**E**) and bright field (**F**).

After 48-h (for bacteria) and 120-h incubation (for archaea) of the above 14 halotolerant microorganisms in the MGL medium, respectively, the fermented cells stained by Nile red were analyzed by fluorescence microscopy. The results showed that only four endogenous microorganisms of the silkworm excrement had significant red fluorescence compared with the genetically engineered PHA-defeated strain ΔEC, namely, the strains A85 (*Haloarcula hispanica*) and A112 (*Natrinema altunense*), BSF4 (*Halomonas salina*), and B6 (*Halomonas janggokensis*) (Figure 1). Further, the PHA accumulation of the above four strains was quantified by gas chromatography, and the results showed that all the PHA accumulating strains initially identified by the Nile red method were able to accumulate the PHAs, but their accumulation did not seem to be linearly related to the light intensity observed by the fluorescence microscopy analysis; among which, the PHA accumulations of the strains A85 (0.68 ± 0.05 g/L), A112 (0.69 ± 0.04 g/L), and BSF4 (0.72 ± 0.02 g/L) were higher and close to each other, while the PHA accumulation of the strain B6 (0.15 ± 0.03 g/L) was significantly lower than that of strains A85, A112, and BSF4 (Table 2).

### 2.3. Effects of Temperature, Salinity, and pH on the Growth of PHA-Producing Strains

**Figure 2:**
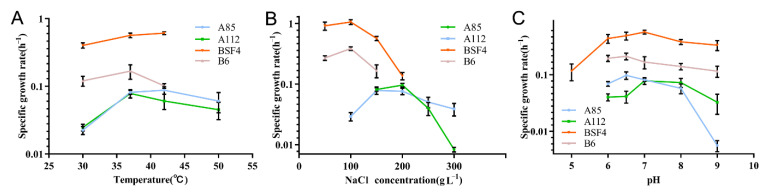
The optimization of the culture conditions for strains A85 (blue line), A112 (green line), BSF4 (orange line), and B6 (pink line). The AS-165 medium was used as the basic medium with specific gradient settings, including: (**A**) temperature (30, 37, 42 and 50 °C); (**B**) salinity (5, 10, 15, 20, 25 and 30%); and (**C**) pH (5, 6, 6.5, 7, 8, and 9). All the data are presented as means of duplicates with their standard errors.

After determining the PHA accumulation capacity of the four strains, we further optimized the culture conditions of the selected strains in terms of the temperature, salinity, and pH. The parameter gradient settings for each condition were described above. The results showed that, when the temperature range was from 30 to 50 °C for the strain A85, the growth conditions in 37 °C and 42 °C appeared to have no significant difference, which was better than that in the other temperatures. The optimal growth temperature for the strain BSF4 was 42 °C, and the optimal growth temperature for the strains A112 and B6 was 37 °C for both (Figure 2A). When the salinity range was from 50 to 300 g/L, the optimum growth salinity of the strain A85 was 200 g/L, strain A112 had the best growth status at salinities of 100 g/L and 150 g/L, strain B6 had an optimum growth salinity of 100 g/L, and strain BSF4 had optimum growth salinities of 50 g/L and 100 g/L (Figure 2B). When the pH range was from 5 to 9, the optimal growth pH was 6.5 for strain A85, 5 and 6.5 for strain B6, 7 and 8 for strain A112, and 7 for strain BSF4 (Figure 2C).

Based on the optimization of the culture conditions for the above four microorganisms, and in order to reduce the cost of industrial fermentation, the optimum growth temperature, salinity, and pH were 37 °C, 200 g/L, and 6.5 for strain A85; 37 °C, 150 g/L, and 7 for strain A112; 37 °C, 100 g/L, and pH 7 for strain BSF4; and 37 °C, 100 g/L, and pH 6.5 for strain B6.

### 2.4. Utilization of Silkworm Excrement by Strains

**Figure 3:**
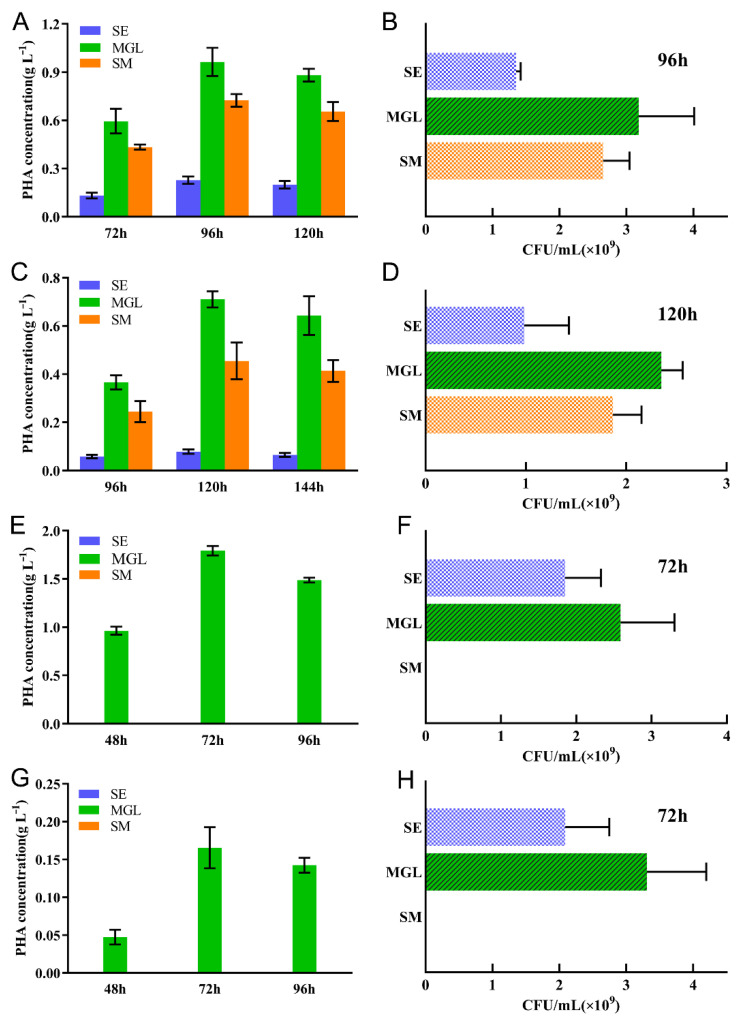
The utilization of the waste silkworm excrement by different strains. The PHA accumulations ((**A**) A85, (**C**) A112, (**E**) BSF4, and (**G**) B6)) were detected by a gas chromatography analysis, and the growth conditions of strains A85 (**B**), A112 (**D**), B6 (**F**), and BSF4 (**H**) determined in the SE (blue), MGL (green), and SM mediums (orange) were indicated by CFU counting. All the data are presented as means of duplicates with their standard errors.

The PHA accumulation of these four microorganisms was tested by using the silkworm excrement as the sole (SE medium) or partial (SM medium) carbon source. Firstly, the results showed that the amount of PHA accumulation was increased after optimization of the culture conditions. Strain A85 had the highest PHA production of 0.96 ± 0.06 g/L with 96 h of fermentation using glucose as the carbon source. Additionally, the PHA production on the SM medium was high, up to 75% of the MGL medium, at 0.72 ± 0.03 g/L. However, the PHA production on the SE medium with the silkworm excrement as the sole carbon source was lower, at 0.23 ± 0.02 g/L. Meanwhile, strain A112 showed the highest PHA production with 96 h of fermentation using glucose as the carbon source at 0.71 ± 0.02 g/L. The PHA production on the SM medium was 0.46 ± 0.05 g/L, up to 65% of the MGL medium. The PHA production by the silkworm excrement as the sole carbon source was 0.08 ± 0.01 g/L (Table 3). Both haloarchaea showed the highest PHA accumulation in the medium with glucose as the sole carbon source and could accumulate PHAs using the silkworm excrement as the sole carbon source. However, in contrast to the haloarchaea, the two bacteria BSF4 and B6 could not accumulate PHAs using the silkworm excrement as the sole or partial carbon source but could use glucose as the carbon source to accumulate PHB, and the highest PHA production was observed with 72 h of fermentation, with 1.79 ± 0.03 g/L for strain BSF4 and 0.17 ± 0.02 g/L for strain B6 (Figure 3A,C,E,G).

Along with the detection of PHA production, we also investigated the growth of the four strains in the silkworm excrement medium using the CFU method. Although two bacteria could not accumulate PHA using the silkworm excrement, their growths seemed to be superior to that of archaea in the SE medium. The CFU values of strains BSF4 and B6 in the SE medium seemed to have no significant difference from those in the MGL medium (Figure 3F,H), but the growths of the two haloarchaea in the MGL medium were significantly better than that of the SE or SM medium (Figure 3B,D). In addition, it was shown that, even in the glucose containing the SM medium, the two bacteria could not grow in the SM medium (Figure 3F,H).

### 2.5. Feasibility Study of the Open Fermentation Process

**Figure 4:**
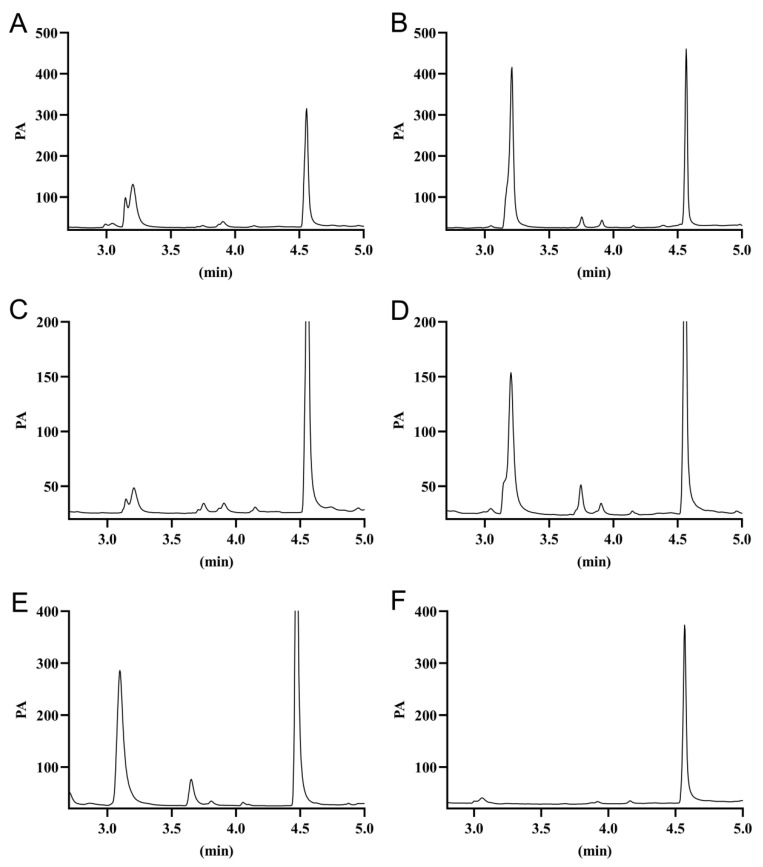
Gas chromatography analysis of PHAs obtained from different cultures of the isolates grown in the unsterilized SE and SM mediums. (**A**) Strain A85 in the SE medium, (**B**) strain A85 in the SM medium, (**C**) strain A112 in the SE medium, (**D**) strain A112 in the SM medium, (**E**) positive control, PHBV standard (Sigma Aldrich Catalog No: 403121, 12 mol % PHV content), and (**F**) negative control, the SE medium without seed culture inoculation. The peaks at 3.2 min and at 3.75 min represent the 3-hydroxybutyrate (3-HB) methyl ester and 3-hydroxyvalerate (3-HV) methyl ester, respectively.

In this study, two haloarchaea, A85 and A112, were tested as the potential candidates in open fermentation using the silkworm excrement as the carbon source due to their significant advantages in terms of yield, salinity, etc. The open fermentation was performed on the unsterilized SE and SM mediums for 72–144 h. The results of the gas chromatography analysis showed that the two haloarchaea could both accumulate PHAs by using the silkworm excrement (Figure 4), and the accumulation of PHAs could not be detected in the natural fermentation of the silkworm excrement medium without seed culture inoculation (Figure 4F). Strain A85 accumulated the highest amount of PHA with 96 h of fermentation in the SM medium, up to 0.81 ± 0.05 g/L and the PHA production of 0.31 ± 0.01 g/L with the silkworm excrement as the sole carbon source; the PHAs production was slightly increased compared to the sterilized fermentation. The highest production of PHAs by strain A112 after 120-h incubation in the SM medium was 0.58 ± 0.04 g/L and 0.08 ± 0.01 g/L in the SE medium; its synthetic amount of PHAs was similar to the sterilized fermentation effect (Table 4). Although the yield of strain A112 was not as high as that of strain A85 in terms of PHA synthesis, it can be seen from the gas spectrum that strain A112 accumulated PHAs with a high 3-HV content of about 13.06% ± 0.03%, while strain A85 accumulated PHAs with a lower 3-HV content of 4.49% ± 0.04% (Figure 4).

**Figure 5:**
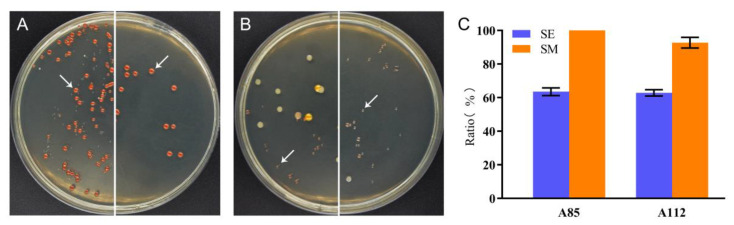
The CFU assay for studying the microbial composition in the open fermentation. The Figure A85 and A112 at the highest PHA yields (4 and 5 days, respectively) were used for the CFU analysis. The left half-circles in (**A**,**B**) were the fermented broths of the SE medium, and the right half-circles were the fermented broths of the SM medium (with the addition of 0.5% glucose). The white arrows indicate target strains A85 (**A**) and A112 (**B**), respectively. More than 20 randomly colonies from each plate were 16S rDNA analysis in the open fermentation. The ratio of the dominated strain was calculated by divided the number of the target colonies to the total number of all chose colonies (**C**). The blue and orange columns indicated the SE medium and SM medium, respectively. All the data are presented as means of duplicates with their standard errors.

To study the microbial community composition in the open fermentations, the fermented medium at the time points of the highest PHA productions (4 and 6 days, respectively) was used for the CFU analysis. More than 20 colonies per sample were randomly selected and sequenced identification (Figure 5A,B). The results showed that four strains of halophilic microorganisms were identified in the SE medium, including strains *Haloarcula hispanica*, *Natrinema altunense*, *Halorubrum cibarium*, and *Gracilibacillus orientalis*; among them, strain A85 (*Haloarcula hispanica*) accounted for 63.50% ± 1.87%, while, in the SM medium, all the colonies tested were strain A85. For strain A112, five halophilic microorganisms were identified in the SE medium, including strains *Natrinema altunense*, *Halorubrum saccharovorum*, *Marinococcus halotolerans*, *Alkalibacillu shalophilus*, and *Bacillus qingdaonensis*, of which strain A112 (*Natrinema altunense*) accounted for 62.83% ± 1.55%; in the SM medium, the number of strains decreased, and only strains *Natrinema altunense* and *Marinococcus halotolerans* were identified, with strain A112 accounting for a higher percentage of 92.73% ± 2.58% (Figure 5C). The above results indicated that the two strains of halophilic archaea had a relatively close ecological advantage of open fermentation, and both were able to maintain about 63% of the colony proportion in the silkworm excrement medium, and it is noteworthy that the addition of glucose significantly enhanced the environmental percentage of the seed microorganisms.

## 3. Discussion

The chlorophyll ethanol-extracted silkworm excrement is a kind of environmental pollutant that is difficult to be reused [6,8]. Two haloarchaea, strains A85 and A112, isolated and identified from the silkworm excrement in this study were shown to convert inexpensive carbon sources to PHAs under high-salt conditions. After optimization of the culture conditions, the two haloarchaea were proven to be able to accumulate PHAs using chlorophyll ethanol-extracted silkworm excrement as the only carbon source, respectively. The possibility of open fermentation of the silkworm excrement by these two haloarchaea was offered by high-salt stress as well.

Previous research of high-salt open fermentation conversion of agricultural waste for PHA production was based on microorganisms of the genus *Halomonas*, including the strain *Halomonas campaniensis* LS21 for PHA production using food waste conversion [22], the utilization of the strain *Halomonas halophila* for food processing waste with carbon source [11], and the open continuous fermentation model study of the strain *Halomonas* TD01 [21], where the strains *Halomonas* TD01 and *Halomonas halophila* have high PHA production. However, in our study, we found that a variety of halophilic bacteria, including the endogenous microorganisms *Halomonas salina* BSF4 and *Halomonas janggokensis* B6, in the silkworm excrement identified in this study; both were unable to accumulate PHAs using the silkworm excrement as the sole or partial carbon source (Figure 3E,G). On the other hand, the haloarchaea A85 and A112 were better able to use the silkworm excrement for PHA production. There are reports of PHA production by halophilic archaea using industrial wastewater, and the halophilic archaea *Natrinema altunense* and *Haloterrigena jeotgali* identified from Chott El Jerid Lake were shown to accumulate PHAs using industrial sugar wastewater, with the strain *Natrinema altunense* being the same strain as A112 in this study. However, in the above study, the accumulation of PHAs was about 0.15 g/L under a 2-g/L glucose addition, while strain A85 in this study could accumulate up to 0.3 g/L of PHAs in the medium with silkworm excrement as the only carbon source (Table 4). The accumulation of PHAs was significantly increased when glucose was added as a partial carbon source, and a certain content of 3-HV component copolymer was accumulated.

In this study, a total of 14 halotolerant bacteria or haloarchaea were isolated, and two halotolerant bacteria and two haloarchaea could accumulate PHAs in a medium with glucose as the carbon source, as confirmed by the Nile red staining and GC assays, but only two archaea showed that they could accumulate PHAs using the silkworm excrement as the only carbon source. The method of Nile red for PHA staining is a common means, but due to its lack of better negative control, the method of Nile red is more likely to produce false-positive results, because the Nile red can also be bound to the cell membrane. In the present work, a deletion mutant strain of PHA synthase, ΔEC, was introduced, and the results showed that it could be used as a suitable negative control to preliminary determine the accumulation of PHAs, and the results were basically consistent, as confirmed by the GC method. Most studies have used PCR with a degenerate primer for the PHA synthase gene as a primary screening method for PHAs accumulating bacteria, but in our study, the accuracy of the PCR method had a much higher false-positive rate than the Nile red staining method [24].

It has been reported that archaea are better able to utilize inexpensive carbon sources compared to bacteria and are able to accumulate PHAs using unrelated carbon sources [16], which is consistent with our results. Most of the above microorganisms grew significantly better in the medium with glucose as the sole carbon source than with silkworm excrement. It is noteworthy that, even containing higher concentrations of glucose, strains BSF4 and B6 could not grow in the SM medium but could grow in the medium with the silkworm excrement as the sole carbon source; it seems that the combination of glucose and silkworm excrement produced an inhibitory factor that hindered the growth of bacteria but did not significantly affect the growth of archaea. This may be related to the physiological metabolic characteristics of haloarchaea, but the exact mechanism has not been elucidated.

The two haloarchaea A85 and A112 accumulated PHAs with different characterizations; strain A85 could accumulate high amounts of PHAs (0.96 ± 0.06 g/L), but its 3-HV content was lower (6.65 ± 0.18%), while strain A112 had a relatively high 3-HV content (15.26 ± 1.01%), although the amount of PHA was low(0.37 ± 0.02 g/L) (Table 3), which might be related to the difference of its 3-HV synthesis-related genes [25]. Among the known archaeal PHA-producing strains, the highest content of 3-HV fraction is the strain *Haloferax mediterranei*, with a maximum content of about 9.33 ± 0.13 mol% [26], and the highest content of strain A112 in this study was found to be 15.26 ± 1.01% in the MGL medium. The halotolerant bacteria isolated in this study that could only accumulate PHB in the MGL medium, which is also in accordance with the reports of related studies.

In this study, two PHA-producing haloarchaea isolated and identified in a high-salt environment conferred the possibility of open fermentation of the silkworm excrement. In a pre-experiment, we tested the microbial growth in silkworm excrement at different salinities with a CFU method [24], and the results showed that, in the 10% NaCl concentration AS-165 agar plates, the number of countable microorganisms per mL of the non-sterilized SE extracts was 2 × 10^5^ CFU/mL, while the number of such endogenous microorganisms detected in 15% NaCl concentration AS-165 plates was substantially lower. The optimum growth salt concentrations of the two halophilic archaea isolated in this study were 15% and 20% NaCl, respectively, which met the salinity requirements for open fermentation in the pre-experiments.

The results of the open fermentation experiments with the medium using the silkworm excrement as the sole carbon source showed that, after a 96-h fermentation, strains A85 and A112 were able to occupy the main ecological dominance (their CFU reached more than 60%), and the addition of glucose had a significant effect on improving the ecological dominance of the two archaea, with strain A85 having almost undetectable symbionts in the end after 5 g/L of glucose addition. In contrast, strain A112 could also increase the percentage of CFU from 62.83 ± 1.55% to 92.73 ± 2.58% (Figure 5C). This result provides an important basis for the optimization of conditions for the subsequent development of fermentation.

In addition, the two archaea did not differ significantly between the non-sterilized and sterilized silkworm excrement mediums, where strain A85 also had a slight improvement, the reason for which has not been able to be elaborated. It may be related to the collaborative symbiosis of microorganisms under carbon source poor conditions; in the open fermentation culture, the symbionts in the fermentation product of strain A85 were mainly strain A112 and *Halorubrum aidingense* and *Gracilibacillus orientalis*, while the symbionts in the fermentation product of strain A112 did not contain PHA-producing strains, mainly *Halorubrum saccharovorum*, *Marinococcus halotolerans*, *Alkalibacillus halophilus*, and *Bacillus qingdaonensis*. Whether open fermentation coculture of the two strains can optimize the current fermentation method still needs further study.

## 4. Materials and Methods

### 4.1. Pretreatment of Silkworm Excrement and Culture Mediums

The chlorophyll ethanol-extracted silkworm excrement used in this study was provided by Fengming Chlorophyll Company Limited (Haining, Zhejiang Province, China). In order to quantify the nutrients in the water-soluble fraction of silkworm excrement, the total sugars and total nitrogen of the solution extracted from silkworm excrement were first determined by the phenol-sulfuric acid method [27] and Kjeldahl nitrogen determination method, respectively [28]. The treatment was as follows briefly: 5 g of chlorophyll ethanol-extracted silkworm excrement sample was shaken with 100 mL of 15% sodium chloride solution at room temperature for 2 h at 200 rpm, and the extracts were obtained by analytical filter paper extraction. The total sugar content was measured as 9.425 ± 0.04 g/L, and the total nitrogen content was 0.88 ± 0.05 g/L. The amount converted to crude protein was about 5.5 ± 0.31 g/L.

The medium used for the isolation and identification of halophilic microorganisms was AS-165 [29]: per liter, 150 g of NaCl, 20 g of MgSO_4_·7H_2_O, 2 g of KCl, 1.2 g of sodium glutamate, 5 mg of FeSO_4_·7H_2_O, 0.036 mg of MnCl_2_·4H_2_O, 3 g of trisodium citrate, 5 g of yeast extract, and 5 g of casamino, pH 7. Add 1.5% agar when needed.

The fermentation medium (MGL medium) for PHA accumulation by halophilic microorganisms contained (per liter) 150 g of NaCl, 9.6 g of MgCl_2_, 14.4 g of MgSO_4_·7H_2_O, 5 g of KCl, 1 g of CaCl_2_, 3 g of yeast extract, 2 g of NH_4_Cl, 0.0375 g of KH_2_PO_4_, 10 g of glucose, 3 g of PIPES, 5 mg of FeSO_4_·7H_2_O, and 0.036 mg of MnCl_2_·4H_2_O, pH 7 [29].

To facilitate a comparison of the effect of the silkworm excrement as a carbon source for microbial fermentation, this study replaced the main carbon source glucose in the MGL medium with a mass of the silkworm excrement of equal carbon content, approximately 53 g/L, named SE. The medium in which the MGL medium was mixed with the SE medium in an equal volume was called SM.

### 4.2. Isolation and Identification of Endogenous Microorganisms in Silkworm Excrement

Each (100 μL) of the silkworm excrement sample previously described was serially diluted in a 15% sodium chloride solution and spread on a 165 medium plate. After incubated at 37 °C for 4–7 days, the colonies with different morphological characteristics were picked up, culture-enriched, and then amplified with bacterial- or archaeal-specific 16S rDNA full-length primers, respectively. The amplified bacterial primer sequences were 27F (5′-AGAGTTTGATCCTGGCTCAG-3′) and 1492R (5′-ATTACCGCGGCTGCTGG-3′), and the archaeal primers were 18F (5′-ATTCCGGTTGATCCTGCC-3′) and 1518R (5′-AGGAGGTGATCCAGCCGC-3′).

The PCR was performed in a 20-μL reaction volume containing 2 × Accurate Taq Master Mix 10 μL (Vazyme), forward primer (100 μmol/L) 1 μL, reverse primer (100 μmol/L) 1 μL, template DNA 1 μL, and ddH_2_O up to 20 μL. The PCR program was as follows: 95 °C for 3 min, 30 cycles of 95 °C for 30 s, 54 °C for 30 s, 72 °C for 90 s, and a final extension 72 °C for 10 min. The PCR products were analyzed by 1.0% agarose gel electrophoresis. The full length 16S rDNA PCR products were cloned into a T-vector (pGEM-T, Promega, USA) and the plasmid isolated from the positive colony were sequenced on an ABI 3730XL platform for further identification. All the strains were stored at −80 °C with 25% glycerol for further study.

### 4.3. Detection of PHA Production Using a Microscopy Approach and Gas Chromatography

The primary screening of PHA-producing halophiles was performed using the Nile red method [30], where the target strains were inoculated into MGL medium and cultured until the end of logarithm or stable phase, harvest 1 mL of culture by centrifugation (60 s, 13,000 rpm), discard the supernatant, add 15% NaCl solution, and resuspend the cell pellet. Each 40 μL of cells were stained by adding 10 μL of Nile red solution (0.1 mg/mL) and incubating for 20 min in a lightproof tube. The cells were observed by a fluorescence microscope (12 V, 4.15A/EVOS FL; Thermo Scientific, Leica DM4; Wetzlar, Germany). To exclude false positives introduced by nonspecific staining of the cell membrane by Nile red, we used a PHA synthase-deficient mutant halophilic strain *Haloferax mediterranei* ΔEC as the negative control; the strain ΔEC was obtained from Xiang’s Lab by Chinese Academy of Sciences (Beijing, China) [26].

The quantification analysis of PHA production of the target strains was performed using the gas chromatography technique (GC) [31]. The fermented cells were harvested by centrifuge and freeze-dried to collect the powder. A certain amount of powder was put into an esterification tube (approximately 80 mg), and 4 mL of esterification solution was added, which was 2 mL of chloroform and 2 mL of 3% (*v/v*) concentrated sulfuric acid in a methanol solution containing benzoic acid (1 g/L), and the esterification reaction was carried out at 100 °C for 4 h. The organic phase was analyzed by gas chromatography using an Agilent Technologies 7890A chromatograph with an injection volume of 1 μL, an inlet temperature of 200 °C and a detector temperature of 220 °C, an initial column temperature of 80 °C, a dwell time of l.5 min, a ramp up to 140 °C at a rate of 30 °C/min, and a ramp down to 140 °C at a rate of 40 °C/min. PHBV standard (Sigma Aldrich, Catalog No: 403121, 12 mol % PHV content) was used as the standard control.

### 4.4. Optimization of Culture Conditions

The seed cultures of the target strain at the late log phase were inoculated as 1:10 into AS-165 medium separately and cultivated at 37 °C, 200 rpm. The AS-165 medium was used as the basic medium for investigating the optimum sodium chloride concentration, PH, and cultural temperature of PHA-accumulating strains with specific gradient settings, including temperature (30, 37, 42, and 50 °C); salinity (5, 10, 15, 20, 25, and 30%); and pH (5, 6, 6.5, 7, 8, and 9). The OD_600_ was used to characterize the growth status of the microorganisms. Finally, the specific growth rates (u) were calculated in the exponential growth period (u = 0.693/td, td: doubling time, h).

### 4.5. Feasibility Study on the Open Fermentation Process

The SE medium without sterilization was used for the study of the possibility of open fermentation of the silkworm excrement, and the accumulation of PHAs was quantified by the GC method under the same conditions as sterilization. The growth of halophiles was done using the CFU method instead due to the interference of the native color of the SE medium. The seed cultures were inoculated into the SE medium at a ratio of 1:10 for fermentation, and at the endpoint of the fermentation, the fermented broth was diluted in an appropriate proportion (10^−5^–10^−7^) and spread on 165 medium plates. More than 20 colonies were randomly picked for 16S rDNA identification. The ratio of the dominated strain was calculated by divided the number of the target colonies to the total number of all chose colonies.

### 4.6. Statistical Analysis

The results in this study were expressed as the means ± SD. Sequence homology was analyzed by the BLAST service (National Center for Biotechnology Information. http://blast.ncbi.nlm.nih.gov/Blast.cgi, accessed on 24 October 2021) in the National Center for Biotechnology Information (NCBI) [32]. Statistical data analysis was performed using the one-way ANOVA method. *p* < 0.05 was considered statistically significant. Three independent experiments were performed for each result.

## Figures and Tables

**Table 1:** Isolation and identification of the endogenous halotolerant microorganisms in silkworm excrement.

Strains		Identity	Strains		Identity
BSF4	*Halomonas salina*	100.00%	BZ7	*Staphylococcus epidermidis*	100.00%
B6	*Halomonas janggokensis*	98.89%	BZ8	*Gracilibacillus orientalis*	100.00%
BZ2	*Oceanobacillus kimchii*	100.00%	BZ9	*Bacillus oryzaecorticis*	100.00%
BZ3	*Halobacillus hunanensis*	100.00%	A11	*Halorubrum saccharovorum*	100.00%
BZ4	*Alkalibacillus halophilus*	100.00%	A28	*Halorubrum aidingense*	99.88%
BZ5	*Marinococcus halotolerans*	100.00%	A85	*Haloarcula hispanica*	99.72%
BZ6	*Brachybacterium paraconglomeratum*	99.72%	A112	*Natrinema altunense*	100.00%

**Table 2:** The PHA accumulation quantified by gas chromatography.

Strains	CDW (g/L)	PHAs Concentration (g/L)	PHAs Content (%)
A85	4.68 ± 0.13	0.68 ± 0.05	14.63 ± 1.51
A112	6.08 ± 0.58	0.69 ± 0.04	11.29 ± 0.46
BSF4	2.19 ± 0.09	0.72 ± 0.02	32.97 ± 2.31
B6	3.06 ± 0.11	0.15 ± 0.03	4.75 ± 0.78

**Table 3:** PHA accumulation of haloarchaeal strains in different carbon source mediums.

		SE		MGL		SM	
Strains	Hours (h)	PHAs Conc. (g/L)	HV Fraction (%)	PHAs Conc. (g/L)	HV Fraction (%)	PHAs Conc. (g/L)	HV Fraction (%)
A85	72	0.13 ± 0.01	n.d.	0.59 ± 0.05	7.12 ± 0.08	0.43 ± 0.01	5.41 ± 0.20
	96	0.23 ± 0.02	n.d.	0.96 ± 0.06	6.65 ± 0.18	0.72 ± 0.03	5.22 ± 0.22
	120	0.20 ± 0.02	n.d.	0.88 ± 0.03	6.61 ± 0.20	0.65 ± 0.04	4.97 ± 0.07
A112	96	0.06 ± 0.01	n.d.	0.37 ± 0.02	15.26 ± 1.01	0.24 ± 0.03	14.45 ± 0.07
	120	0.08 ± 0.01	n.d.	0.71 ± 0.02	15.00 ± 0.18	0.46 ± 0.05	14.31 ± 0.98
	144	0.07 ± 0.01	n.d.	0.64 ± 0.06	14.17 ± 0.35	0.41 ± 0.03	13.77 ± 0.73

n.d.: not detectable.

**Table 4:** The PHA accumulation in open fermentation.

		SE		SM	
Strains	Hours (h)	PHAs Conc. (g/L)	HV Fraction (%)	PHAs Conc. (g/L)	HV Fraction (%)
A85	72	0.16 ± 0.01	n.d.	0.49 ± 0.03	4.52 ± 0.13
	96	0.31 ± 0.01	n.d.	0.81 ± 0.05	4.49 ± 0.04
	120	0.29 ± 0.02	n.d.	0.76 ± 0.06	4.05 ± 0.14
A112	96	0.08 ± 0.01	n.d.	0.30 ± 0.02	14.59 ± 0.30
	120	0.12 ± 0.02	n.d.	0.58 ± 0.04	13.06 ± 0.03
	144	0.11 ± 0.01	n.d.	0.50 ± 0.03	13.50 ± 0.17
control		n.d.	n.d.	n.d.	n.d.

n.d.: not detectable.

## Data Availability

The data presented in this study are available in article.

## Sample Availability

Samples of polyhydroxyalkanoates are available from the authors.
